# Utilizing Cardiac POCUS for the Diagnosis of Dynamic Mitral Regurgitation

**DOI:** 10.24908/pocusj.v11i01.19963

**Published:** 2026-04-22

**Authors:** Sheila Wee, Suneil K. Aggarwal, David Hoare, Olusegun Olusanya

**Affiliations:** 1 Adult Critical Care Unit, Royal London Hospital, Barts Health NHS Trust, London, UK; 2 Department of Anaesthesiology and Intensive Care, Khoo Teck Puat Hospital, NHG Health, Singapore; 3 Department of Cardiology, Barts Heart Centre, Saint Bartholomew's Hospital, Barts Health NHS Trust, London, UK; 4 Department of Perioperative Medicine, Barts Heart Centre, Saint Bartholomew's Hospital, Barts Health NHS Trust, London, UK

**Keywords:** POCUS, Critical care, Mitraclip, Point of Care Ultrasound, Echocardiography, Value-driven care

## Abstract

We report a case of an elderly gentleman with recurrent episodes of flash pulmonary edema, initially attributed to hypertensive episodes. Cardiac point of care ultrasound (POCUS) revealed severe mitral regurgitation (MR), a finding underappreciated on transthoracic echocardiography and likely responsible for his repeated episodes of deterioration. Early recognition of dynamic MR expedited and guided targeted management of his valvular pathology. This case report highlights how serial cardiac POCUS can complement transthoracic echocardiography.

## Introduction

Dynamic mitral regurgitation (MR) is challenging to diagnose, as severity may vary significantly according to loading conditions [1]. Thus, it can be difficult to accurately recognize and assess on routine transthoracic echocardiograms. Serial cardiac point of care ultrasound (POCUS) examinations can address this diagnostic gap, capturing a transient phenomenon during symptomatic episodes [2]. Proper training with advanced cardiac POCUS techniques is required [3–5].

## Case Presentation

A 69-year-old man with a history of cerebrovascular disease, type 2 diabetes mellitus, hypertension, and hyperlipidaemia presented to the emergency department (ED) with sudden onset acute shortness of breath for 2 hours. He then deteriorated rapidly within 10 minutes of arrival to the hospital and suffered a witnessed cardiac arrest with return of spontaneous circulation after 4 minutes of resuscitation. He was intubated and sedated.

A 12-lead electrocardiogram showed new left bundle branch block. Cardiac and lung POCUS showed poor left ventricular contractility and bilateral B-lines pattern, respectively. Laboratory investigations revealed the first Troponin T level was 55 ng/L, which subsequently rose to 18,415 ng/L (reference range <14 ng/L). There were severe respiratory and metabolic acidosis and hypoxemia with a pH of 6.67, pCO_2_ of 9.69 KPa, pO_2_ of 11.5 KPa (FiO_2_ 1.0), bicarbonate of 8 mmol/L and lactate of 10.3 mmol/L. The chest X-ray was consistent with fluid overload. The impression was acute pulmonary edema from a non-ST-elevation myocardial infarction.

On the second day of admission, a transthoracic echocardiogram demonstrated the left ventricle to be normal in size with impaired systolic function (ejection fraction of 45%). There were multiple regional wall motion abnormalities with akinesia of all the apical segments and the mid-anteroseptal segment. The remaining walls were contracting normally. The mitral valve appeared structurally normal with mild MR.

Over the course of 2 days, fluid removal was initially achieved via haemodialysis and thereafter with furosemide. He was extubated on day 2 and transferred to the general ward on day 6 of admission with plans for an inpatient coronary angiogram.

**Figure 1.**
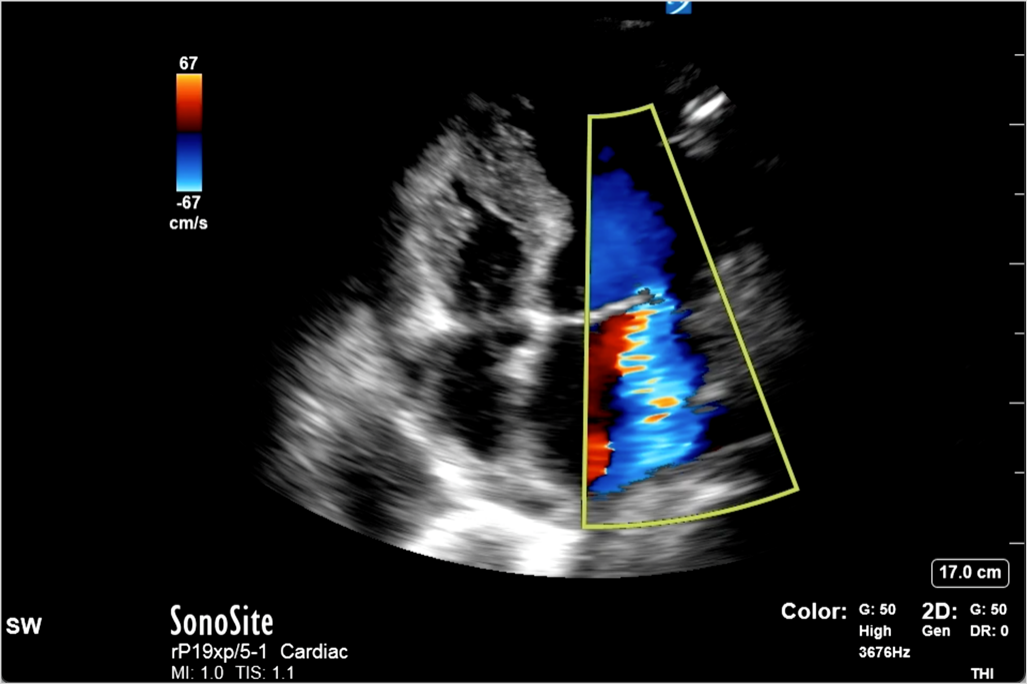
Cardiac point of care ultrasound (POCUS), apical four chamber view, demonstrating thin mitral valve leaflets and a large mitral regurgitant jet occupying half of the left atrium and extending to the posterior wall of the left atrium on color Doppler imaging.

On the general ward, there was a repeat episode of flash pulmonary edema. He was receiving oxygen at 2 L/min when his oxygen saturation rapidly declined to 50%, requiring a 100% non-rebreather mask. He was subsequently re-intubated and mechanically ventilated. Repeat cardiac POCUS demonstrated left ventricular impairment and regional wall motion abnormalities similar to the recent transthoracic echocardiogram. It further showed a significant MR jet that was posteriorly directed, occupying 50% of the left atrium and reaching the left atrial wall (Figure 1).

He was stabilized again and transferred to a cardiac center, where he was extubated on their intensive care unit. He continued to have recurrent episodes of flash pulmonary edema related to increases in heart rate and blood pressure. These episodes were managed with inotropic support, diuretics, and continuous positive airway pressure (CPAP). A repeat transthoracic echocardiogram showed impaired left ventricular function but only mild MR. This led to the presumption that the episodes were purely afterload related. There were large bilateral pleural effusions noted; drainage of the right-sided pleural effusion was performed with minimal improvement in symptoms. The pleural fluid was transudative in nature according to Light's criteria [6].

A coronary angiogram revealed severe diffuse disease in the left anterior descending coronary artery, critical in mid-vessel, and proximal-mid right coronary artery disease and further posterior descending artery disease. Medical therapy was recommended as no stents were placed.

On day 10 of admission, cardiac POCUS was repeated as part of teaching rounds. Prior to the examination, the patient's blood pressure was 80/50 mmHg with a heart rate of 70 beats/min, supported by milrinone and adrenaline infusions. During the POCUS examination, his blood pressure rose to 110/60 mmHg and his heart rate increased to 90 beats/min as he became increasingly aggravated while sharing his frustrations about his prolonged hospitalization. Interestingly, the posteriorly directed mitral regurgitant jet occupying 50% of the left atrium was seen again, which was visually quantified as severe by the supervising intensive care unit consultant (Supplementary Material S1).

Based on his history, lab findings, and review of the POCUS findings, a multidisciplinary team comprising cardiologists, intensivists, and cardiothoracic surgeons concluded that the patient had dynamic MR, which was the leading cause of his deterioration. In view of his medical background, both percutaneous and surgical interventions for his coronary artery disease were deemed extremely risky, and the recommendation was for ongoing medical optimisation for his coronary artery disease. Mitral valve intervention in the form of transcatheter edge-to-edge repair with MitraClip™ (Abbott, 3200 Lakeside Dr, Santa Clara, 95054, U.S.A) was discussed with and accepted by the patient.

The patient was transferred to the cardiac catheterization laboratory, anaesthetised, and had a baseline transesophageal echocardiogram (TEE) performed. This demonstrated moderate-severe secondary MR due to tethering of the posterior mitral valve leaflet (Supplementary Material S2). Two clips were successfully placed under fluoroscopic and TEE guidance, and the patient was stepped down to the general ward 4 days post procedure, where guideline directed medical therapy for heart failure was optimised. There were no further episodes of flash pulmonary edema.

## Discussion

Advanced cardiac POCUS examination with Doppler assessment of the mitral valve was pivotal in diagnosing dynamic MR that was underappreciated on initial transthoracic echocardiogram [7,8]. Secondary MR remains a challenge to diagnose, as it is characteristically dynamic in nature and sensitive to changes in ventricular geometry and loading conditions [9]. In diseased ventricles, the delicate balance of tethering and closing forces that is necessary for optimal mitral valve leaflet coaptation is often disrupted, resulting in MR. Both volume overload and increase in afterload, provoked either physiologically or pharmacologically, can result in further changes in left ventricular geometry and hence, worsening of MR [10]. Therapies for secondary MR should thus aim at reducing the dynamic component of the valvular lesion [9].

Current guidelines recommend modalities such as TEE, exercise stress echocardiography, cardiac magnetic resonance imaging and cardiac computed tomography to complement two-dimensional echocardiography in the evaluation of MR. These are not without risk or additional cost or time; particularly in our patient who was CPAP and inotrope-dependent. In patients whose clinical presentation is not fully explained by their resting echocardiography findings, exercise stress echocardiography is recommended to elicit severe MR [11]. This is, however, not practical in critically ill patients. In such a setting, the worsening on the MR can be provoked either physiologically or pharmacologically via vasopressors or fluid infusion.

In this case, POCUS enabled the team to identify findings which only became evident in situations where the patient was hemodynamically stressed. This facilitated a more rapid diagnosis and allowed for timely intervention, which could potentially translate to a shorter length of stay and reduced healthcare costs [12].

## Conclusion

This case demonstrates that advanced cardiac POCUS can hasten the diagnosis of dynamic MR. Without POCUS, this patient might have required further investigations such as a repeat transthoracic echocardiogram or TEE for diagnosis, which would have resulted in significant delays.
